# South West Orthopoedic Club, November Meeting 1989

**Published:** 1990-09

**Authors:** 


Southmead Hospital, Bristol

### South West Orthopaedic Club

### Meeting held at Bristol Royal Infirmary, Saturday 4th November, 1989

The meeting consisted of clinical presentations in the morning and a Guest Lecture by Mr Ian Watt, Consultant Radiologist, Bristol Royal Infirmary, who talked about the place of Magnetic Resonance Imaging in Orthopaedics.

The South West Orthopaedic Club Prize was presented to Mr M F Gargan for his Paper entitled "The Long Term Prognosis of Soft Tissue Injuries of the Neck", which was work done when he was a Registrar at Southmead, although he presently is a Registrar in Oxford. There was a *Proxime accessit* award to Miss D M Eastwood of Bristol. Her Paper was entitled "Trigger Fingers–The Direct Approach".

### AUTOLOGOUS BLOOD TRANSFUSION AND TOTAL KNEE ARTHROPLASTY

**R. S. Majkowski, I. Currie, J. H. Newman**
Winford Orthopaedic Hospital

A prospective randomised trial was undertaken to evaluate autologous blood transfusion using the Solcotrans orthopaedic system to collect blood drained following total knee replacement. We were particularly interested in:

1) Savings in the use of bank blood.
2) The safety and practicality of this system.

Forty patients having unilateral total knee arthroplasty were entered into the trial (20 Solcotrans, 20 control). There were 28 women and 12 men (average age 70.8 yrs).

The mean total drainage in the two groups was 1138 mls (control) and 1020 mls (Solcotrans) ($P>0.1$). Homologous blood was required in 19 out of 20 patients (95%) in the control group (mean 2.5 units per patient) but in only seven out of 20 (33%) in the Solcotrans group (mean 0.9 units per patient) ($P<0.001$), a saving of 1.6 units per patient. A mean of 470 mls per patient of autologous blood was reinfused in the Solcotrans group.

Haemoglobin was measured pre-operatively intra-operatively (prior to release of tourniquet), and at 1, 4 and 8 days post-operatively. Haemoglobin on admission was 12.7 in the control group and 13.2 in the Solcotrans group ($P>0.1$). At eight days it was 11.6 and 11.4 respectively ($P>0.1$). There were no complications arising specifically from the use of this system or reinfusing autologous blood. A significant reduction in the need for bank blood has been demonstrated.

### "THE TECHNIQUE AND CLINICAL RESULTS OF A DOUBLE-OBLIQUE PLANAR TIBIAL OSTEOTOMY"

**T. P. Green**
Gloucestershire Royal Hospital

The use of high tibial osteotomy in osteoarthritis of the knee is well described and a widely practiced operation. The procedure corrects progressive varus deformity and reduces pain in correctly selected cases. Various methods have been described, the commonest methods being either a closing wedge or a dome osteotomy.

We describe a different type of osteotomy, it being a planar cut that relies on it being oblique in 2 planes to produce its effect. No bone is removed and the osteotomy allows for tibial tuberosity elevation and the correction of tibial torsion where necessary.

The technique has been used successfully at Gloucester for the last 4 years. In terms of pain relief, range of movements and walking ability the double-oblique osteotomy is shown to be comparable in effect to the other more widely used techniques with the benefit of being a simpler procedure.

### THE PALMAR CUTANEOUS BRANCH OF THE MEDIAN NERVE; THE RATIONALE FOR VOLAR WRIST INCISIONS BASED ON THE ANATOMY

**P. A. Magnusen**
Bristol Royal Infirmary
**R. J. Hobbs, M. A. Tonkin**
Royal North Shore Hospital, Sydney, Australia.

The palmar cutaneous branch of the median nerve was dissected in 25 fresh cadaveric forearms and hands. Previous studies by Carroll, Taliesnik and Bezerra have dissected this nerve but only formalin-preserved specimens. The origin, course and distribution of terminal branches were defined with a reference to two standard lines. One, at the level of the distal wrist crease and the second bisecting this line in the line of the axis of the middle finger. The PCN was on average 12.9 cm in length with 4.5 cm of this distal to the wrist crease. There were on average 4 branches to the radial side distal to the crease averaging 1.6 cm in length. On average there were 2 branches from the ulnar side of the PCN averaging 0.4 cm in length. The maximum distance from the distal wrist crease was 6 cm, the maximum distance ulnar to the mid-axial line 1 cm, and radial to this line 4 cm. These figures differ from the previous cadaveric dissections and, we believe, are more representative of the true situation because of the ease of dissection in the fresh specimens.

As a result of these anatomical findings recommendations are made for the planning of surgical incisions around the volar aspect of the hand and wrist, i.e. carpal tunnel surgery, approaches for the volar wrist ganglion and the volar approaches to both the distal radius and scaphoid.

### DIASTASIS OF THE PUBIC SYMPHYSIS FOLLOWING CHILDBIRTH

**M. W. Scriven, Dai Anthony Jones**
Morriston Hospital, Swansea SA6 6NL

Diastasis of the pubic symphysis usually results from trauma. After childbirth it may arise spontaneously or as a result of surgical symphysiotomy. Post mortem studies have found features of mechanical damage in the pubic symphysis to be a consistent finding in women delivered vaginally.

A series of eight women who had spontaneous diastasis of the pubic symphysis following childbirth, is reported. The clinical features presented a stereotyped syndrome which was easily identified if the condition was borne in mind.

Plain radiology was undertaken to confirm the diagnosis in most cases. It added little to the management, which was conservative in all cases. In those that presented during the course of the study, CT and ultrasound scanning were undertaken.

Follow up of the women indicated that the conservative management was justified. However information on long term problems is lacking and in its absence decisions regarding the best form of management must be limited.

Reported incidence has ranged widely. This series indicates that the condition is under diagnosed.

A prospective study is being undertaken in Swansea to investigate the problem further and to assess the role of CT and ultrasound scanning in its management.

### THE LONG-TERM PROGNOSIS OF SOFT TISSUE INJURIES OF THE NECK

**M. F. Gargan and G. C. Bannister**
Southmead Hospital, Bristol

Soft tissue injuries of the cervical region following rear end collision can be troublesome and the long-term prognosis difficult.

Sixty-three such patients presented to the Bristol Royal Infirmary between 1977 and 1980 and 64% were symptomatic after 2 years. Forty-three of these were reviewed 10 years after injury and 7 years after settlement of litigation. Residual symptoms and the range of neck movement were recorded.

The commonest symptoms were neck pain (74%), paraesthesia (45%), thoracolumbar pain (42%), headache (33%) and vertigo (19%). Twelve per cent of patients were symptom-free and in 48% they were a nuisance but did not interfere with activity. Twenty-eight per cent were limited in their work and recreation and required frequent intermittent analgesia, orthotic support or physiotherapy. Twelve per cent were severely affected, had lost their jobs, took regular analgesia and wore orthoses continually. Older patients were more severely affected. The range of neck movement was equally restricted amongst all grades of disability.

Hyperextension injuries of the neck do not appear to resolve with time. The inexact correlation of symptoms with physical signs might suggest psychological overlay but their persistence long after settlement of litigation and their specific association with one direction of trauma favours an organic lesion.

### THE ROLE OF SURGERY IN THE MANAGEMENT OF METASTATIC LESIONS OF THE CERVICAL SPINE

**Mr. C. Weatherley, Dr. L. Vanden Berghe, Dr. H. Mehdian**
Princess Elizabeth Orthopaedic Hospital, Exeter

Failure to adequately treat metastatic lesions of the cervical spine has disastrous consequences for the patient. The role of surgery and the techniques available remain subjects for discussion.

This paper reports different surgical techniques used in the management of metastatic lesions of the cervical spine. Three cases will be described, each of different aetiology and all with a progressive neurological involvement.

The role and timing of surgery will be discussed together with other lessons learnt from these different cases.

### OBLIQUE OSTEOTOMY WITH SCREW FIXATION FOR HALLUX VALGUS

**R. D. Case, R. Z. Taylor and C. E. Ackroyd**
From Southmead Hospital, Bristol and Medical City Dallas, Texas

A horizontal oblique osteotomy of the neck of the first metatarsal is performed parallel to the plane of the sole of the foot. The metatarsal head is displaced laterally and stabilized with an interfragmentary screw. Bunionectomy and medial capsular reefing are then performed.

This operation was used for adolescent metatarsus primus varus and elderly hallux valgus (ages 14–72) with hallux valgus angles of 19–90 degrees and intermetatarsal angles of 7–22 degrees. The study was prospective and the results of the first 90 patients were analysed at two years. Forty patients had bilateral operations producing 130 toes to analyse. The results were assessed by patient satisfaction, foot function and radiological measurements.

Overall 114 (88%) of feet were judged satisfactory by the patient at two year review. Sixty-five (52%) were back at work or walking well at 4 weeks and by 8 weeks this had increased to 112 (90%) of cases. One-hundred-and-eighteen (91%) cases were able to stand all day and had a normal gait. One-hundred-and-twenty (92%) of cases were relieved of their bunion pain and 33 (27%) no longer had metatarsalgia. One-hundred-and-eleven (85%) of toes were straighter. Sixty-four (52%) of feet had an improvement in hallux valgus angle of 10 degrees or more and 98 (86%) of feet had a decrease in their intermetatarsal angle.

The AO cancellous screw had to be removed in 49 (38%) of cases due to pressure on the skin. In five toes a second operation was necessary, one to curette a deep infection, one to arthrodese a stiff metatarsophalangeal joint, one was transformed to a Keller's following a non-union and in two cases the initial deformity was only partially corrected at the initial operation.

A superficial infection was present in 27 (20%). In 22 (17%) this settled within four weeks and in the rest by eight weeks. The one deep infection settled after curettage and removal of the screw.

The oblique osteotomy creates a large area of contact of cancellous bone which is compressed by weightbearing and by lag screw fixation. The screw holds the corrected position and allows early weight-bearing without a plaster. Patient satisfaction is high (88%) and the number of complications compares favorably with that following other operations.

### TRIGGER FINGERS: THE DIRECT APPROACH

**D. M. Eastwood and D. P. Johnson**
Bristol

Stenosing tenosynovitis of the finger is a relatively common affliction. By the time it causes actual triggering of the finger by interfering with free tendon excursion during finger movements, the "nuisance" value is high and the patient's hand function is compromised. We have redeveloped a technique which involves the percutaneous release of the A1 pulley of the flexor tendon sheath using a standard 21G hypodermic needle and is suitable for use in patients who have palpable triggering of the finger. The procedure is performed under local anaesthetic in the out patient department.

Twenty-four patients have been treated by this method. In 23 cases the treatment has been successful. In 1 patient the technique failed and an open release was performed at a later date. No significant complications have occurred.

Many of these patients have waited several months to be seen in the orthopaedic outpatient clinic and it is rewarding for the surgeon to be able to relieve their condition quickly, comfortably and reliabiy at their initial attendance.

